# MCM-22, MCM-36, and ITQ-2 Zeolites with Different Si/Al Molar Ratios as Effective Catalysts of Methanol and Ethanol Dehydration

**DOI:** 10.3390/ma13102399

**Published:** 2020-05-22

**Authors:** Monika Marosz, Bogdan Samojeden, Andrzej Kowalczyk, Małgorzata Rutkowska, Monika Motak, Urbano Díaz, Antonio E. Palomares, Lucjan Chmielarz

**Affiliations:** 1 Faculty of Chemistry, Jagiellonian University, Gronostajowa 2, 30-387 Kraków, Poland; monika.skoczek@doctoral.uj.edu.pl (M.M.); kowalczy@chemia.uj.edu.pl (A.K.); rutkowsm@chemia.uj.edu.pl (M.R.); 2 Faculty of Energy and Fuels, AGH University of Science and Technology, Mickiewicza 30, 30-059 Kraków, Poland; motakm@agh.edu.pl; 3 Instituto de Tecnología Química, Universitat Politècnica de València—Consejo Superior de Investigaciones Científicas, Avd. de los Naranjos s/n, 46022 Valencia, Spain; udiaz@itq.upv.es (U.D.); apalomar@iqn.upv.es (A.E.P.)

**Keywords:** MCM-22, MCM-36, ITQ-2, methanol, ethanol, dehydration

## Abstract

MCM-22, MCM-36, and ITQ-2 zeolites with the intended Si/Al molar ratios of 15, 25, and 50 were synthetized and tested as catalysts for dehydration of methanol to dimethyl ether and dehydration of ethanol to diethyl ether and ethylene. The surface concentration of acid sites was regulated by the synthesis of zeolite precursors with different aluminum content in the zeolite framework, while the influence of porous structure on the overall efficiency of alcohol conversion was analyzed by application of zeolitic materials with different types of porosity—microporous MCM-22 as well as microporous-mesoporous MCM-36 and ITQ-2. The zeolitic samples were characterized with respect to their: chemical composition (ICP-OES), structure (XRD, FT-IR), texture (N_2_ sorption), and surface acidity (NH_3_-TPD). Comparison of the catalytic activity of the studied zeolitic catalysts with other reported catalytic systems, including zeolites with the similar Si/Al ratio as well as γ-Al_2_O_3_ (one of the commercial catalysts for methanol dehydration), shows a great potential of MCM-22, MCM-36, and ITQ-2 in the reactions of alcohols dehydration.

## 1. Introduction

Increasing concerns about the climate change, energy security and independence, as well as diminishing oil resources drive the search for new alternative energy sources, including fuels based on renewable raw materials. Such new generation of fuels could be biomethanol and bioethanol and ethers obtained by their dehydration—dimethyl ether (DME) and diethyl ether (DEE), respectively. Biomethanol and bioethanol are chemically identical to conventional methanol and ethanol but are produced from renewable feedstock and therefore the usage of fossil fuel resources can be reduced. Biomethanol, also called renewable methanol, is produced by gasification of virgin or waste biomass into syngas, which after conditioning to reach the optimal CO_2_/H_2_ ratio, is catalytically converted to methanol. The cost of biomethanol production is estimated to be 1.5 and 4 times higher than the cost of natural gas-based methanol and strongly depends on the type of feedstock used [1,2,3]. Bioethanol is manufactured by fermentation of biomass, especially waste biomass. After fermentation, alcohol is separated by distillation of fermented broths. Distillation is an energy consuming operation and accounts for the overall cost of bioethanol production. Therefore, the optimization of the distillation of fermented broths is one of the most important challenges in this technology [4]. Among the possible uses of biomethanol and bioethanol, their dehydration to DME and DEE, is gaining a great deal of attention.

DME, due to properties similar to liquefied petroleum gas (LPG), is used as a domestic fuel blended with LPG [5]. Moreover, the use of DME as a substitute for LPG and household cooking fuel has been reported [6]. DME is used as diesel fuel additive due to its relatively high cetane number (about 55–60), high oxygen content (34.8% by mass) and lack of C-C bond [7,8,9]. Therefore, DME combustion results in low emissions of nitrous oxides and particulate matter compared to diesel fuel combustion [10,11]. Moreover, DME is non-toxic, non-corrosive as well as can be easily liquefied and transported and therefore has gained great attention in the transportation sector. Dehydration of ethanol at lower temperatures results mainly in DEE, while at higher temperatures in ethylene. DEE is characterized by very good properties of transportation fuels (cetane number above 125) [12]. Combustion properties of DEE make it a very promising alternative fuel or diesel fuel additive [13]. It was reported that blending of DEE with ethanol significantly improved the cold start in ethanol fueled cars [14]. Therefore DEE, similarly to DME, is a chemical of great potential for the transportation sector.

Synthesis of DME and DEE from methanol and ethanol, respectively, is reported to be via acid catalyzed, exothermic reactions. In both reactions, solid acid materials were reported to be catalytically active. Commercial catalysts used for methanol to DME dehydration are among others—zeolites (HY or HZSM-5), γ-Al_2_O_3_, silica-alumina, or phosphorus-alumina [8,9,15,16,17]. On the other hand zeolites, heteropoly acids, modified clay minerals and other solid acid materials were reported as active catalysts of ethanol to DEE dehydration [12,18,19,20,21]. Thus, surface acidity is very important for effective conversion of alcohols to ethers.

The main goal of the studies was comparison of three series of the zeolitic catalysts—MCM-22, MCM-36, and ITQ-2—with different surface acidity, regulated by the Si/Al ratio, in the reactions of methanol and ethanol dehydration. The studied zeolites are characterized by various porous structures—MCM-22 is microporous material, MCM-36 is silica intercalated layered zeolite with the bi-modal microporous and mesopores structure, while ITQ-2 is layered delaminated material with the microporous-mesoporous structure. Thus, another goal of the study was showing the influence of the porous structure of zeolites on their catalytic efficiency in alcohols dehydration. The reaction products—dimethyl ether and diethyl ether—significantly differ in the molecular size and therefore differences in the overall reaction rate, related to the internal diffusion limitations, could be expected.

## 2. Experimental

### 2.1. Zeolite Synthesis

MCM-22(P)—precursor of MCM-22, MCM-36, and ITQ-2—was synthesized according to the procedure reported by Corma et al. [22]. To prepare the reactant gel, 0.675 g sodium aluminate (Carlo Erba, Val de Reuil, France) and 0.375 g sodium hydroxide (Scharlau, Barcelona, Spain) were dissolved in 80.71 g distilled water (Milli-Q). Then 4.96 g fumed silica (Aerosil 200, Evonik, Essesn, Germany) and 6.00 g hexamethyleneimine (HMI, Merck, Darmstadt, Germany) were added into the solution. The obtained mixture was stirred for 2 h at room temperature and then transferred to 30 mL Teflon-lined stainless-steel autoclaves. After aging at 130 °C for 7 days (60 rpm) the samples were removed from autoclaves. The obtained product, MCM-22(P), was washed with distillated water until the pH decreased to about 7, then the sample was filtered and dried at 60 °C overnight. This procedure was applied to obtain the MCM-22(P) sample with the Si/Al molar ratio of 15. Similar procedures, but with different reactant proportions, were used for the synthesis of the MCM-22(P) sample with the intended Si/Al molar ratios of 25 and 50. The obtained MCM-22(P) sample was used for the synthesis of MCM-22, MCM-36, and ITQ-2 zeolites.

MCM-22 was obtained from dried MCM-22(P) by its calcination at 580 °C for 3 h in air (with the ramps at 150 °C for 2.5 h and 350 °C for 3 h). During calcination organic surfactants were removed from MCM-22(P) together with the condensation of zeolite layers and the formation of 3D microporous structure of MCM-22. As result, three samples of this series—MCM-22_15, MCM-22_25, and MCM-22_50—with the intended Si/Al molar ratios of 15, 25 and 50, respectively, were obtained.

To prepare silica intercalated MCM-36 as well as delaminated ITQ-2 zeolites, MCM-22(P) has to be swollen, by dispersion of 10 g of the lamellar precursor in 40 g of H_2_O milliQ, and 200 g of a cetyltrimethylammonium hydroxide solution (25 wt %, 70% exchanged Br^−^/OH^−^) and 60 g of a solution of tetrapropylammonium hydroxide (40 wt %, 70% exchanged Br^−^/OH^−^) were added, being the final pH ≥ 12.5. The obtained slurry was heated at 80 °C, stirring vigorously, for 16 h to facilitate the swelling of the layers in the precursor material.

Intercalation of swollen MCM-22(P) with the silica pillars results in MCM-36 zeolite. In the first step, swollen MCM-22(P) was mixed with tetraethyl orthosilicate (TEOS, 98%, Merck, Darmstadt, Germany) with the ratio of 1:5 (wt/wt). The obtained mixture was stirred at 80 °C for 24 h in dinitrogen atmosphere. Then, the solid product was separated by filtration, washed with ethanol and acetone, and dried overnight at 60 °C. In the next step dried, modified zeolite was dispersed in distilled water (Milli-Q) with the ratio of 1:10 (wt/wt) and stirred at 80 °C for 24 h. After washing with distilled water and drying overnight at 60 °C, the samples were calcined at 540 °C for 1 h in dinitrogen atmosphere and then for 6 h in air atmosphere resulting in the MCM-36 zeolite. Depending on the intended Si/Al molar ratios in MCM-22(P) zeolites used for the synthesis, three samples of the MCM-36 series were obtained—MCM-36_15, MCM-36_25, and MCM-36_50 with the intended Si/Al molar ratios in the zeolite layers of 15, 25, and 50, respectively.

The ITQ-2 samples were synthetized according to the procedure described in [23]. The second part of swollen MCM-22(P) was converted into delaminated ITQ-2 zeolite. The slurry of the swollen MCM-22(P) sample was sonicated in an ultrasound bath (50 W, 40 kHz) for 1 h. Then, the pH of the mixture was decreased to 2 by addition of hydrochloric acid (37%, Merck, Darmstadt, Germany), separated by centrifuging (12,000 rpm, 15 min), washed with distilled water to obtain pH = 7 and dried overnight at 60 °C. Finally, the samples were calcined at 540 °C for 1 h in dinitrogen atmosphere and then for 6 h in air atmosphere, resulting in ITQ-2 zeolite. Three zeolites of ITQ-2 series with the intended Si/Al molar ratios of 15 (ITQ-2_15), 25 (ITQ-2_25), and 50 (ITQ-2_50) were synthetized.

### 2.2. Characterization of Catalysts

The chemical analysis of the zeolitic samples were done by inductively coupled plasma optical emission spectroscopy method—ICP-OES (iCAP 7400, Thermo Scientific, Waltham, MA, USA). In the first step, the zeolitic samples were dissolved in a solution of hydrofluoric, sulfuric, and phosphoric acids assisted by microwave radiation (Ethos Easy, Milestone). The X-ray diffraction (XRD) patterns of the samples were collected with D2 PHASER powder diffractometer (Bruker, Billerica, MA, USA). The diffractograms were taken using Cu-Kα radiation (λ = 1.54184 Å) in the 2θ range of 3–70° with a step of 0.02° and a counting time of 1 s per step. The average crystallite size of MCM-22 was estimated by the analysis of the full width at half maximum (FWHM) of the (1 0 0), (1 0 1) and (1 0 2) diffraction peaks. Textural parameters of the samples were determined by N_2_ adsorption–desorption measurements at −196 °C using an ASAP 2010 (Micromeritic, Norcross, GA, USA) instrument. Prior to the analysis, the zeolitic samples were outgassed under vacuum at 350 °C for 24 h. The specific surface area (SSA) was estimated using BET model, while total pore volume was calculated assuming the total amount of adsorbed dinitrogen at p/p_0_ = 0.98.

The concentration of surface acid sites and their relative acid strength was analyzed by the method of temperature-programmed desorption of ammonia (NH_3_-TPD). NH_3_-TPD studies were done using a flow microreactor system equipped with quadrupole mass spectrometer (QMS) detector (PREVAC, Rogow, Poland). Prior to the NH_3_-TPD runs, the zeolitic samples were outgassed at 500 °C for 30 min in a flow of pure helium. In the next step ammonia was adsorbed on the samples at 70 °C in a flow (20 mL/min) of gas mixture containing 1.0 vol % NH_3_ in helium. Ammonia desorption runs were carried out in a flow of pure helium (20 mL/min) with the linear heating rate of 10 °C/min.

### 2.3. Catalytic Tests

The reactions of methanol dehydration and ethanol dehydration were studied in a flow fixed-bed microreactor system operating under atmospheric pressure. The catalyst (0.1 g) was placed into quartz microreactor on the quartz wool plug and outgassed at 500 °C for 30 min in a flow of pure helium. The reaction mixture composing of alcohol (3.9 vol % of methanol or 3.3 vol % of ethanol) diluted in helium was supplied into microreactor with the flow rate of 20 mL/min. The methanol and ethanol content in the reaction mixture was determined by their volatility at 0 °C (saturation temperature).

The catalytic runs were carried out in the range from 100 °C to 300 °C with isothermal ramps every 25 °C. K-type thermocouple, placed in quartz capillary inside microreactor in the catalyst bed, was used for temperature measuring. Gas chromatograph (SRI 8610C) equipped with methanizer and FID detector was used for the analysis of the reaction mixture before and after microreactor. The operating temperature of chromatography column, depending on the reaction, was 120 °C for methanol dehydration or 180 °C for ethanol dehydration. The results of three chromatographic analyses, done for each isothermal ramp, were averaged.

## 3. Results and Discussion

**Figure 1:**
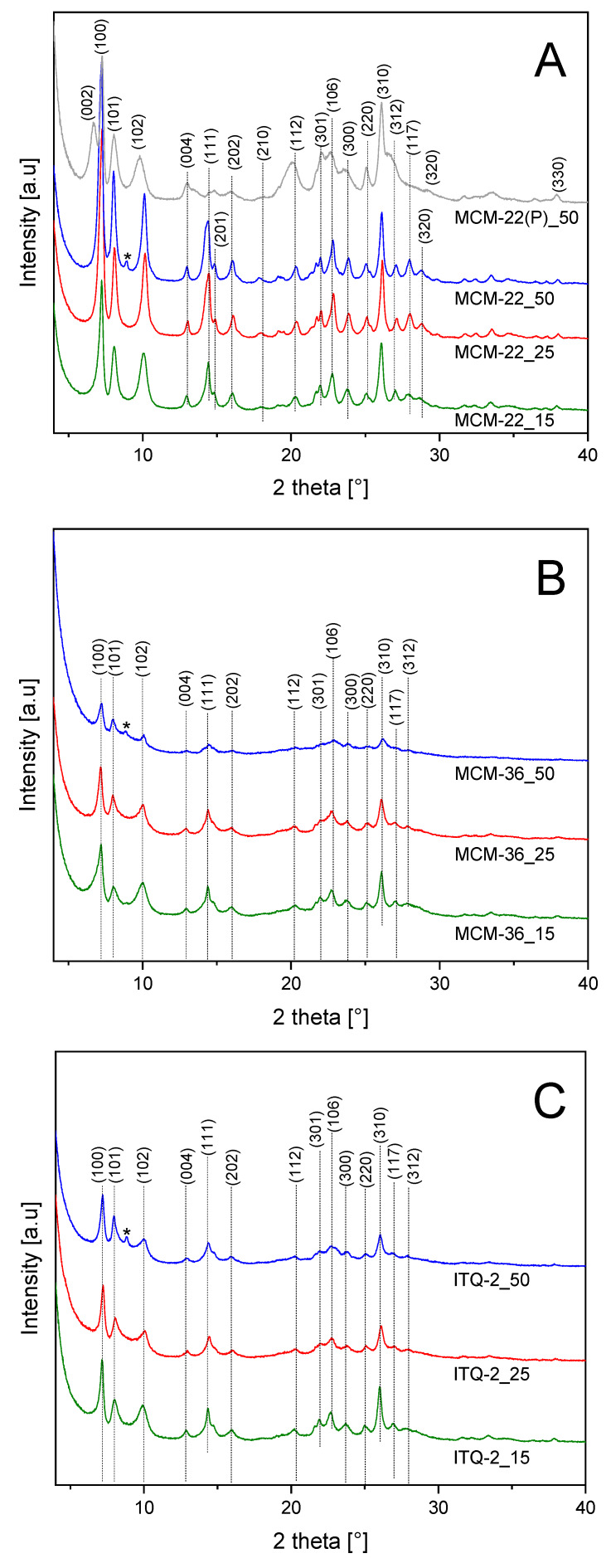
X-ray diffractograms of MCM-22 (**A**), MCM-36 (**B**) and ITQ-2 (**C**) zeolites, * additional silica-alumina phase.

Diffractograms of the zeolitic samples of MCM-22, MCM-36, and ITQ-2 series are presented in Figure 1. Diffraction patterns recorded for the MCM-22 zeolites were compared with diffractogram of MCM-22(P)_50—precursor of the MCM-22_50 sample (Figure 1A). The (0 0 2) reflection at 6.6°θ, indicates the ordered layered structure with the d-spacing of 2.6 nm. Calcination of MCM-22(P) resulted in thermal removal of interlayer templates and condensation of the zeolite layers with the formation of microporous 3D structure. Therefore, in the calcined samples of MCM-22 series the inter-layer reflection (0 0 2) overlaps with an intra-layer reflection (1 0 0), proving the formation of 3D zeolite. Diffractograms recorded for the MCM-22 samples contain reflections characteristics of this type of the zeolite structure [24]. Intensity of the reflections decreased with a decrease in the Si/Al ratio, indicating the formation of the less ordered zeolite structure for the alumina-rich samples. Intercalation of MCM-22(P) with silica pillars, resulting in MCM-36, decreased intensity of the diffraction peaks characteristic of the MCM-22 structure (Figure 1B). This effect is related to the ordered 2D structure, which is limited only to the zeolite layers of MCM-36 in contrast to MCM-22 with the 3D zeolitic structure. The most significant decrease in the reflection intensity was observed for MCM-36_50, showing that the samples with the higher aluminum content are less effectively intercalated than zeolites with the lower alumina content. It is possible that the samples with larger alumina content, due to greater charging of the zeolite layers, are less susceptible to swelling and intercalation. The leak of (0 0 2) reflection in diffractograms of the MCM-36 samples is related to spatial modification of the zeolite layers ordering due to pillarization or delamination processes, which result in the loss of perpendicular order with the respect to c axis. Also, in the case of the ITQ-2 samples (Figure 1C) the intensity of the reflections significantly decreased comparing to MCM-22. However, intensity of these reflections was higher than in diffractograms of the MCM-36 samples, indicating that the obtained ITQ-2 samples are not fully delaminated. The analysis of the average MCM-22 crystallite sizes, <D_XRD_> shows that such crystallites are also present in the samples of MCM-36 and ITQ-2 series (Table 1). The size of these crystallites in these zeolites is significantly smaller than in the series of MCM-22 zeolites. Thus, not all zeolite layers are separated by silica pillars or delamination in MCM-36 and ITQ-2 zeolites, respectively. Apart from the reflections characteristic of MCM-22, additional peak at 8.8° (marked by asterix) is present in diffractograms of all zeolites obtained from MCM-22_50(P). Thus, the additional phase, represented by this reflection, was possibly formed during the synthesis of MCM-22_50(P). The identification of the phase based only on one diffraction peak is very speculative; however, it could be suggested that this reflection is assigned to the small contribution of ZSM-5. First of all, ZSM-5, has the two most intensive reflections exactly at 8.8° and about 8.0° (possibly overlapped with the (1 0 1) reflection of MCM-22). The third strongest diffraction peak of ZSM-5 should be at about 23° and therefore may be overlapped by (1 0 6) reflection of MCM-22. Secondly, the synthesis of ZSM-5 with using hexamethylene imine, used also in the synthesis of MCM-22(P), was reported in scientific literature [25]. However, as it was already mentioned, this hypothesis is very speculative and additional studies should be done to explain this scientific problem.

**Figure 2:**
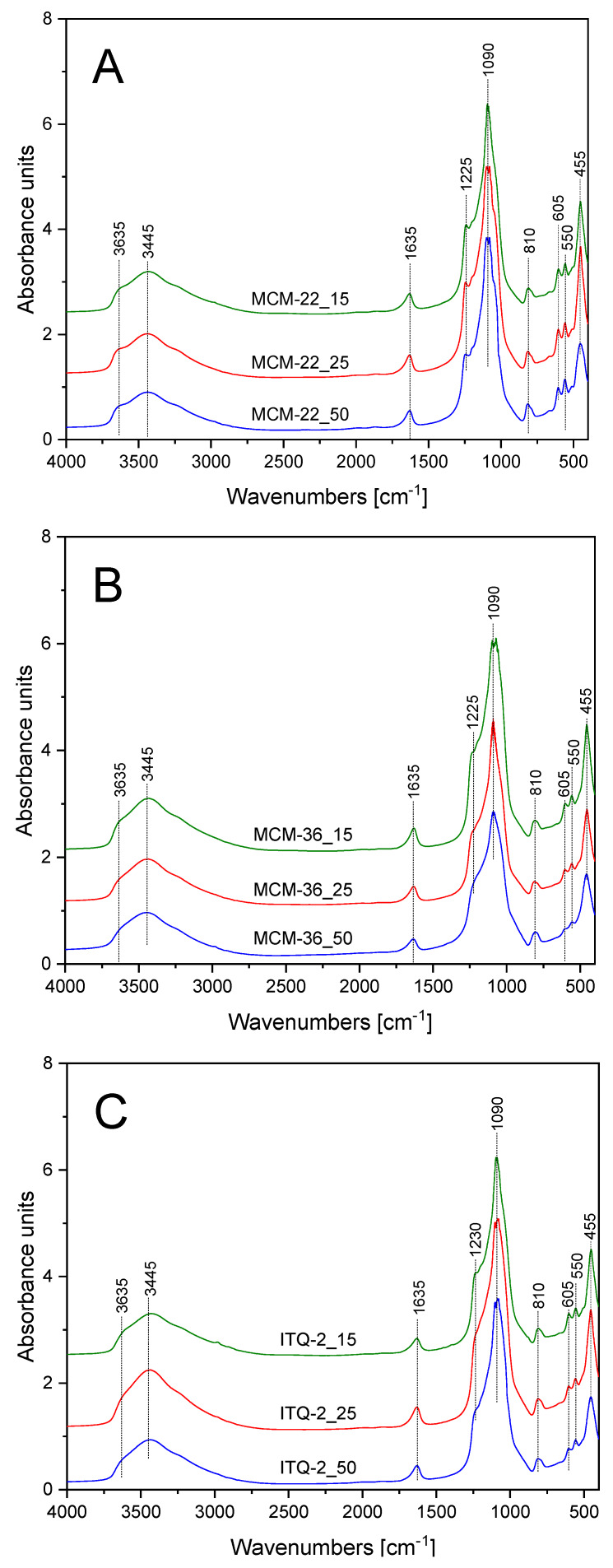
FT-IR spectra of MCM-22 (**A**), MCM-36 (**B**), and ITQ-2 (**C**) zeolites.

FT-IR spectra of the zeolite samples are presented in Figure 2, while the assignment of the characteristic bands is shown in Table 2. Spectra recorded for all the samples are very similar. The main difference is related to higher intensity of the band at about 1225 cm^−1^, assigned to stretching, asymmetric, and symmetric vibrations of T-O-T (where: T = Si and Al) for the MCM-22 series (Figure 2A) comparing to MCM-36 (Figure 2B) and ITQ-2 (Figure 2C). It could be explained by the 3D structure of MCM-22 and therefore the larger number of the ≡T-O-T≡ bridges comparing to MCM-36 and ITQ-2 with the layered structure. Decreased intensity of the bands at 455 and 550 cm^−1^, characteristic of Al-O-Si deformation and O-Al-O blending vibrations, in the samples of the layered MCM-36 and ITQ-2 zeolites, in contrast to MCM-22 zeolites with 3D structure, are possibly related to differences in their structural ordering. Moreover, for the layered MCM-36 and ITQ-2 zeolites intensity of these bands decreased with an increase in the Si/Al molar ratio indicating that delamination and intercalation of MCM-22(P) is more effective for the samples with the lower aluminum content. It is in full agreement with the results of XRD studies.

The real Si/Al molar ratios determined by ICP-OES, as well as textural and surface acidity parameters of the zeolitic samples are shown in Table 1. In all cases, the real Si/Al molar ratios are smaller than the intended ones, which indicates that aluminum cations were more preferably incorporated into the zeolite framework comparing to silicon. Similar results were reported in scientific literature [31,32]. An increase of the Si/Al ratio in MCM-36 series in comparison to the MCM-22 samples is due to intercalation of interlayer silica pillars. On the other hand, an increase of the Si/Al ratio in ITQ-2 is due to the treatment of MCM-22(P) with hydrochloric acid to promote exfoliation of the layered zeolite structure.

**Figure 3:**
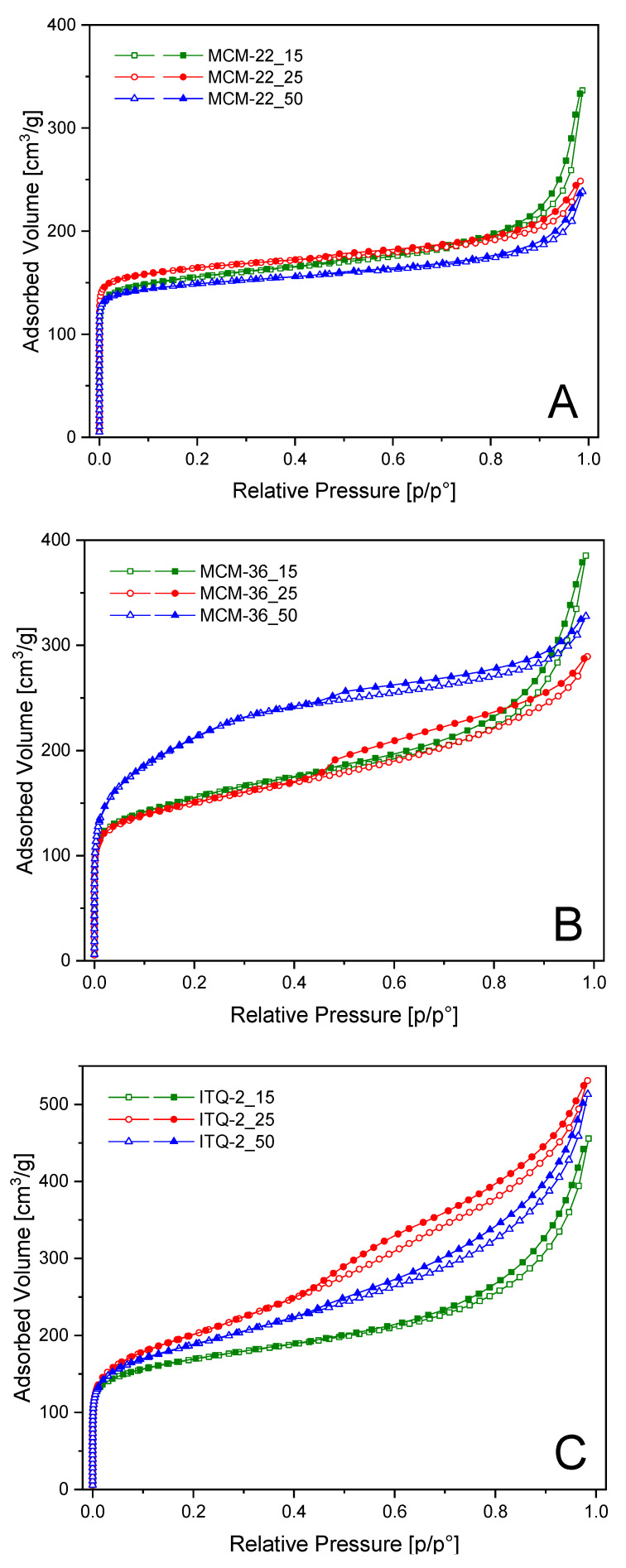
Dinitrogen adsorption–desorption isotherms of MCM-22 (**A**), MCM-36 (**B**), and ITQ-2 (**C**) zeolites.

Dinitrogen adsorption–desorption isotherms of the MCM-22 samples, presented in Figure 3A, are classified as type I, typical of microporous materials. This type of isotherm shows a steep adsorption at low relative pressure, assigned to dinitrogen condensation in micropores [32]. The specific surface area (SSA) determined for the MCM-22 samples is in the range of 584–643 m^2^ g^–1^, total pore volume in the range of 0.369–0.521 cm^3^·g^−1^ with a significant contribution of micropores. Dinitrogen adsorption–desorption isotherms of MCM-36_15 and MCM-36_25, presented in Figure 3B, are similar to isotherms of the MCM-22 samples. However, a gradual increase in dinitrogen adsorbed volume observed above p/p_0_ = 0.02, which is more distinct than for the samples of the MCM-22 series, indicates the presence of larger pores (mesopores and macropores) of non-uniform size. The isotherm of MCM-36_50 has a different profile, especially in the p/p_0_ range of 0.02–0.3, showing significant contribution of mesopores. This sample is characterized by the largest SSA in the series of the MCM-36 samples (Table 2). Thus, the obtained results show that intercalation of silica pillars into MCM-22(P) with the lower alumina content was significantly more effective comparing to the samples with the higher alumina loading. The ITQ-2 samples show a hybrid-type isotherm, that comprised of type I and IV (Figure 3C), characteristic of this type of porous materials, indicating that the micropore structure is still maintained after the partial delamination process. The SSA of the ITQ-2 samples is in the range of 621–720 m^2^·g^−1^ and the total pore volume is significantly larger than for other series of the studied samples. It should be also noted that micropore volume in this series is lower than in other zeolitic samples, indicating effective opening of the interlayer space.

**Figure 4:**
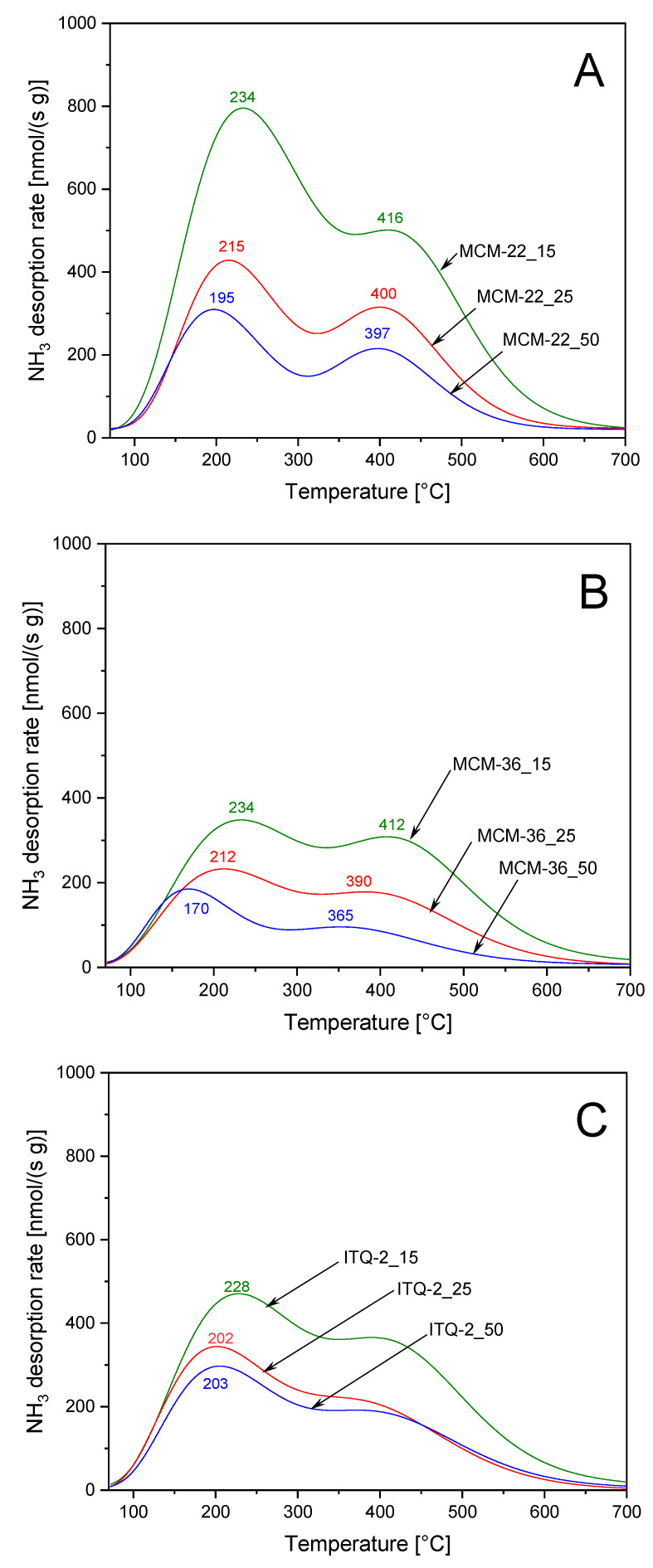
NH_3_-TPD profiles of MCM-22 (**A**), MCM-36 (**B**), and ITQ-2 (**C**) zeolites.

The surface acidity of the zeolite samples was analyzed by temperature-programmed desorption of ammonia (NH_3_-TPD). Ammonia desorption profiles of the MCM-22 series, shown in Figure 4A, consist of two maxima, indicating two types of acid sites of different strength. Low-temperature, centered at about 195–234 °C, is related to ammonia desorption from acid sites of lower acid strength, while the less intensive, high-temperature maximum, centered at about 397–416 °C, is assigned to ammonia desorption from stronger acid sites. The quantity of chemisorbed ammonia is correlated with the content of aluminum, which acts as a source of acidity in zeolites. An increase in aluminum content in the samples of MCM-22 series resulted not only in an increase of the surface concentration of acid sites but also increased acidic strength of these sides. This effect is manifested by the shift of the low- and high-temperature ammonia desorption maxima into higher temperatures with an increase in aluminum content in the samples. Intercalation of MCM-22(P) with silica pillars, resulting in the series of the MCM-36 samples, reduced intensity of ammonia desorption profiles (Figure 4B). This effect is related to introduction of amorphous silica aggregates (interlayer pillars) with no acidity into zeolite. Correlation between aluminum content and acid sites concentration as well as their strength is also observed also for the series of MCM-36 zeolites. The intercalation of zeolite with silica pillars resulted in more effective decrease in the content of the sites with the lower acidic strength. The ammonia desorption profiles of ITQ-2 zeolites also consist of two maxima, assigned to acid sites of various strength (Figure 4C). The concentration of acid sites depended on the alumina content in the samples but the shift in the ammonia desorption maxima for higher temperatures, indicating an increase in acid sites strength, is observed only for the sample with the highest aluminum content—ITQ-2_15.

The studies of the acid sites nature in MCM-22, MCM-36, and ITQ-2 with the intended Si/Al molar ratio of 15 by using FT-IR analysis of the pyridine adsorbed samples were presented and discussed in our previous paper [24]. It was shown that both Brønsted and Lewis acid sites are present in the studied zeolite samples. Brønsted acid sites (BAS) dominated over Lewis acid sites (LAS) and the BAS/LAS ratios determined at 150 °C for MCM-22, MCM-36, and ITQ-2 were 3.32, 2.64, and 1.62, respectively. Thermal treatment of the samples at 350 °C resulted mainly in desorption of pyridine from Brønsted acid sites, indicating lower strength of this type of acid sites comparing to the Lewis type of acid sites. The ratio of pyridine chemisorbed on BAS/LAS for MCM-22, MCM-36, and ITQ-2, thermally treated at 350 °C, were 2.38, 2.02, and 1.20, respectively [24]. Thus, it seems that low-temperature ammonia desorption maxima (Figure 4) are related mainly to ammonia desorbing from weaker Brønsted acid sites. Surface acidity (SA, number of acid sites in 1 g of the sample) and surface density of acid sites (DA, number of acid sites on 1 m^2^ of the sample surface) determined for the studied samples are compared in Table 2. It was assumed that one ammonia molecules is chemisorbed on one acidic site, thus the number of chemisorbed ammonia molecule is equal to the number of acid sites.

**Figure 5:**
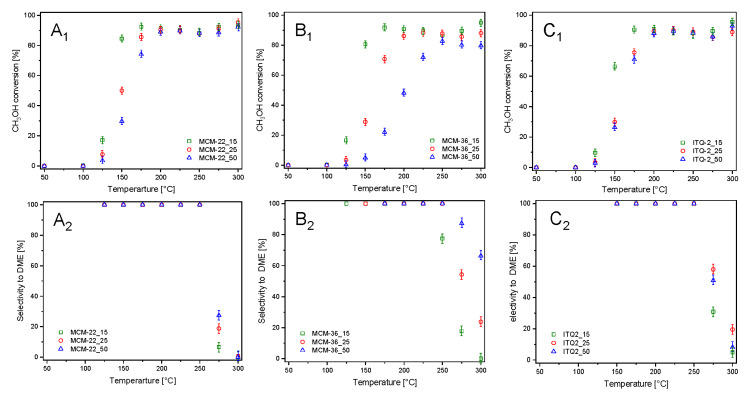
Results of catalytic dehydration of methanol in the presence of MCM-22 (**A_1_**,**A_2_**), MCM-36 (**B_1_**,**B_2_**), and ITQ-2 (**C_1_**,**C_2_**) zeolites.

Results of catalytic studies of methanol to dimethyl ether (DME) dehydration are presented in Figure 5. For the series of MCM-22 catalysts the methanol to DME conversion started at about 100 °C and increased to 175–200 °C, reaching about 89–92%, then up to 250 °C the level of methanol conversion was nearly constant and above this temperature small increase in CH_3_OH conversion was observed (Figure 5A_1_). The incomplete methanol conversion in the range of 175–250 °C is assigned to the thermodynamic restrictions, as methanol dehydration is a slightly exothermic reaction and therefore an increase in the reaction temperature shifts the free reaction enthalpy to higher values, what results in the reaction equilibrium lowering [33]. The selectivity to DME of 100% was up to 250 °C (Figure 5A_2_). An increase in methanol conversion and decrease in the selectivity to DME, observed above 250 °C, are related to the formation of formaldehyde, carbon monoxide and methane, which are the side reaction products. Catalytic activity of the MCM-22 samples strongly depended on aluminum content, indicating a very important role of surface acidity in methanol dehydration. The methanol conversion profile of MCM-22_15 is shifted into lower temperatures by about 25 °C in relation to MCM-22_50. Selectivity to DME decreased with the increasing aluminum content in the samples. Within the MCM-36 series of the samples more significant differences in catalytic activity were observed (Figure 5B_1_). In this case the methanol conversion profile of MCM-36_15 is shifted into lower temperatures by about 65 °C in comparison to MCM-36_50. For the most active catalyst of this series, MCM-36_15, maximal methanol conversion on the level about 91% was obtained at 175 °C. For MCM-36_25 maximal methanol conversion of 88% was achieved at about 225 °C, while in the case of MCM-36_50, the conversion of 82% was obtained at 250 °C. At temperature above 200 °C, the selectivity to DME decreased more intensively due to the formation of the side reaction products, mainly formaldehyde, carbon monoxide and methane, for the samples with the higher aluminum content (Figure 5B_2_). Thus, also in this series of the samples an increased surface acidity resulted in higher catalytic activity and reduced high-temperature selectivity to DME. Also, in the case of the ITQ-2 series, the sample with the highest aluminum content, ITQ-2_15, presented better catalytic activity at lower temperatures comparing to zeolites with the lower aluminum content, ITQ-2_25 and ITQ-2_50 (Figure 5C_1_). In this case the methanol conversion profile of ITQ-2_15 is shifted into lower temperatures by about 15–20 °C in relation to other catalysts of this series. Methanol conversion profiles of ITQ-2_25 and ITQ-2_50 are very close each to other due to a very similar aluminum content and therefore also surface acidity (cf. Table 1). DME was formed with the selectivity of 100% up to 250 °C (Figure 5C_2_), while at higher temperatures the selective to DME drastically dropped down due to the formation of the side reaction products—mainly formaldehyde, carbon monoxide, and methane.

**Figure 6:**
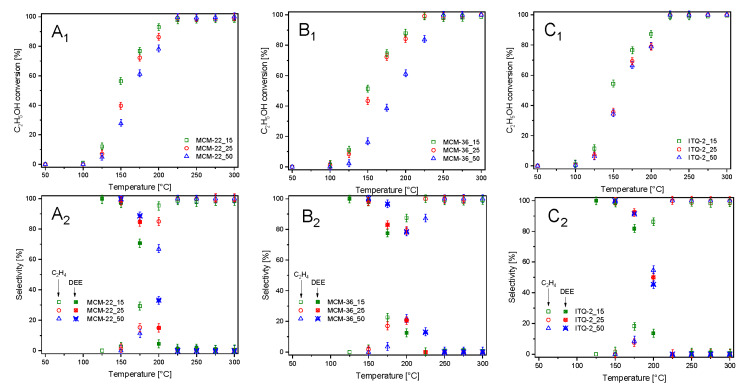
Results of catalytic dehydration of ethanol in the presence of MCM-22 (**A_1_**,**A_2_**), MCM-36 (**B_1_**,**B_2_**), and ITQ-2 (**C_1_**,**C_2_**) zeolites.

The zeolitic samples were also studied as catalysts for ethanol dehydration (Figure 6). In this case the main reaction products are diethyl ether (DEE) and ethylene. Due to thermodynamical restrictions, DEE is produced at lower temperatures, while ethylene is the main reaction product at elevated temperatures. Moreover, at higher temperatures, the formation of small amount of acetaldehyde, carbon monoxide, methane, ethane, and C_3_ hydrocarbons was observed. For all series of the zeolitic catalysts—MCM-22, MCM-36, and ITQ-6—ethanol conversion stared at about 100 °C and increased to 225–250 °C, reaching 98–100% (Figure 6A_1_–C_1_). For all series of the catalysts, the correlation between the content of aluminum and catalytic activity in ethanol conversion was observed, indicating a very important role of the surface acidity in this reaction. However, the shift of ethanol conversion profiles of the catalysts with various aluminum contents is less significant compared to these same catalysts tested in methanol dehydration. Among the studied samples, the best catalytic activity in ethanol dehydration presented MCM-22_15 (Figure 6A_1_), so the sample with the highest surface concentration of acid sites (Table 2). However, the samples of other series with the high aluminum content, MCM-36_15 (Figure 6B_1_) and ITQ-2 (Figure 6C_1_), were only slightly less catalytically active. As it was already mentioned, DEE is thermodynamically favored product of ethanol dehydration at lower temperatures, while ethylene at higher temperatures [21]. For all catalysts, the selectivity to DEE started to decrease at temperature 125–150 °C and at about 225–250 °C dropped to 0% (Figure 6A_2_–C_2_). The selectivity to ethylene changed exactly the opposite direction. It was shown that an increase in aluminum content resulted in decreasing DEE selectivity and increasing ethylene selectivity at lower temperatures. Thus, surface acidity influenced not only the catalytic activity in ethanol conversion, but also selectivity to the reaction products.

The turn-over-frequency values (TOF), determined for the reactions of methanol and ethanol dehydration at 150 °C, are compared in Table 3. It was assumed that all acid sites, determined by NH_3_-TPD method (Table 1), play a role of catalytically active centers. For all series of the studied catalysts, the most active sites of methanol conversion were present in zeolites with the highest content of aluminum, MCM-36_15, MCM-22_15, and ITQ-2_15. Decrease in the aluminum content resulted in decreased activity of catalysts. The results of NH_3_-TPD studies (Figure 4) show that an increase in the aluminum content caused not only an increase of the acid site concentration but also the formation of stronger acid sites (shift of ammonia desorption peaks in direction of higher temperatures). Thus, it could be suggested that stronger acid sites are more active in the reaction of methanol dehydration than weaker sites. The most significant difference in the positions of the ammonia desorption maxima was observed for the MCM-36 series: for low-temperature maximum at 234 °C and 170 °C and for the high- temperature maximum at 412 °C and 365 °C for MCM-36_15 and MCM-36_50 (Figure 4). Also, for these two catalysts the most significant difference in TOF values, 5.4·10^−3^·s^−1^ for MCM-36_15 and 1.0·10^−3^·s^−1^ for MCM-36_50, were found. In our previous paper, a dominating role of Brønsted acid sites in methanol dehydration was postulated [21]. The possible reaction mechanisms include the formation of surface methoxy group (-OCH_3_) by the reaction of the surface Brønsted acid site (≡Si-O(H)-Al≡) with methanol molecule. In the next step such surface methoxy group reacts with another methanol molecule, resulting in the formation of DME. As it was already mentioned, in our previous paper [21] Brønsted acid sites dominated over Lewis acid sites in the MCM-22, MCM-36, and ITQ-2 with the Si/Al molar ratio of 15. Thus, also in the case of these studies an important role of Brønsted type of acid sites is postulated.

TOF values determined for ethanol dehydration at 150 °C (Table 3) are in the range of 2.3·10^−3^–3.1·10^−3^·s^−1^. In contrast to methanol dehydration, there is not any correlation found between TOF’s and strength of acid sites. Thus, it seems that in this case the strength of acid sites is less important than in the case of methanol conversion. On the other side, TOF values determined for dehydration of ethanol over the catalysts of MCM-22 series are lower than for the MCM-36 and ITQ-2 samples. Therefore, the influence of internal diffusion restrictions, more significant in microporous MCM-22 than micro-mesoporous MCM-36 and ITQ-2, on the overall reaction rate—especially in the case of larger DEE molecules—cannot be excluded. The suggested mechanisms of the ethanol to DEE conversion include reaction of alcohol molecule with Brønsted acid sites resulting in the surface ethoxyl group (-OC_2_H_5_), which reacts with another ethanol molecule resulting in DEE. The mechanisms of ethanol to ethylene conversion include direct interaction of the ethanol hydroxyl group with the Brønsted acid site, resulting in the surface ethoxide species. In the next step, the involvement of Lewis basic site is necessary to the proton elimination from surface ethoxide to obtain ethylene [34,35,36,37]. Thus, ethanol to ethylene conversion needs not only Brønsted acid sites but also Lewis basic sites.

Temperatures needed to obtain 50% of methanol and ethanol conversions in the presence of the catalysts studied by authors are shown in Table 4. Such a comparison is possible because in all cases the catalytic tests were done in these same conditions. As it can be seen, dehydration of methanol and ethanol occurred at significantly lower temperatures over zeolitic catalysts comparing to modified minerals and mesoporous silica as well as γ-Al_2_O_3_, which is one of the commercial catalysts for methanol dehydration [21]. The selectivity to DME determined at temperature of 50% methanol conversion is 100%, with the exception of two mineral based samples, allophane and V-2 (Table 4). In the case of ethanol dehydration, the selectivities to DEE are significantly lower and decrease with an increase in temperature of 50% of ethanol conversion, which is in full agreement with thermodynamical limitations of this reaction. In a group of zeolites with the similar Si/Al molar ratio, the studied catalysts—MCM-22_15, MCM-36_15, and ITQ-2_15—were the most active in the low-temperature range. Thus, the studied zeolites seem to be very promising as potential catalysts for methanol and ethanol dehydration.

## 4. Conclusions

Zeolite MCM-22 and its layered modifications, MCM-36 and ITQ-2, with different molar Si/Al ratios and porous structures were synthetized and tested in the role of catalysts for methanol and ethanol dehydration. Catalytic activity of the studied zeolitic samples in the reaction of methanol dehydration was dependent mainly on the surface concentration of acid sites. Moreover, it was shown that the stronger acid sites are more catalytically active compared to weaker sites. The increase in aluminum content in the zeolite frameworks resulted in an increased contribution of stronger acid sites. In the case of ethanol dehydration, apart from surface concentration and strength of acid sites, also the porous structure of the zeolitic samples influenced their overall catalytic performance in this process. It is possibly related to different size of methanol and ethanol molecules, but especially product of their conversion—dimethyl ether and diethyl ether molecules—and their limited rate of internal diffusion in micropores. Thus, the open porous structure, containing both micro- and mesopores, is important only for dehydration of ethanol and should be taken into account in designing effective catalysts for this process.

The zeolitic catalysts presented very good catalytic properties in both studied catalytic processes. The most active catalysts of the MCM-22, MCM-36, and ITQ-2 series were found to be significantly more active than γ-Al_2_O_3_, which is one of the commercial catalysts of methanol dehydration. Moreover, comparison of the results of the catalytic tests performed in these same experimental conditions—obtained for the studied catalysts and other zeolites, Y and ZSM-5 zeolites with the similar Si/Al molar ratio—shows very promising catalytic properties of the high alumina MCM-22, MCM-36, and ITQ-2 samples in methanol and ethanol dehydration.

## Figures and Tables

**Table 1:** Si/Al molar ratio, textural, and acidic properties of the zeolitic samples.

Sample	Si/Al (mol/mol)	SSA (m^2^/g)	V_TOTAL_ (cm^3^/g)	V_MICRO_ (cm^3^/g)	SA (μmol/g)	DA (μmol/m^2^)	<D_XRD_> (nm)
MCM-22_15	11	597	0.52	0.20	1202	2.0	18.1
MCM-22_25	20	643	0.38	0.23	736	1.1	20.2
MCM-22_50	29	584	0.37	0.19	508	0.9	22.6
MCM-36_15	14	561	0.60	0.18	869	1.5	12.3
MCM-36_25	22	549	0.45	0.16	713	1.3	13.5
MCM-36_50	40	761	0.51	0.21	269	0.3	11.5
ITQ-2_15	11	621	0.71	0.18	943	1.5	12.7
ITQ-2_25	18	720	0.82	0.08	537	0.7	13.1
ITQ-2_50	20	680	0.79	0.15	530	0.8	14.8

**Table 2:** Assignments of FT-IR bands in the spectra of zeolitic samples.

Wavenumber (cm^−1^)	Vibrations	References
455	T-O bending (where: T = Si and Al)	[26]
550	Al-O-Si deformation	[26,27]
605	O-Al-O blending	[27]
810	O-Si-O stretching	[27,28]
1090	stretching modes of internal T-O bonds in TO_4_ tetrahedra (where: T = Si and Al)	[26,27]
1225–1230	stretching, asymmetric and symmetric, of T-O-T (where: T = Si and Al)	[27]
1635	-OH deformation	[28]
3445	H-O-H stretching (adsorbed water)	[29]
3550	hydrogen bonded SiO-H groups (a broad band)	[29,30]
3635	-Si-O(H)-Al-, vibration of OH bridging between Al and Si atoms in the zeolite framework	[27,29]

**Table 3:** Turn-over-frequency determined for methanol and ethanol dehydration over zeolitic samples at 150 °C.

Sample	TOF CH_3_OH/150 °C (10^−3^·s^−1^)	TOF C_2_H_5_OH/150 °C (10^−3^·s^−1^)
MCM-22_15	4.1	2.3
MCM-22_25	3.9	2.6
MCM-22_50	3.4	2.7
MCM-36_15	5.4	2.9
MCM-36_25	2.3	3.0
MCM-36_50	1.0	3.0
ITQ-2_15	4.1	2.9
ITQ-2_25	3.2	3.1
ITQ-2_50	2.9	3.1

**Table 4:** Comparison of various catalysts in methanol and ethanol dehydration.

Catalyst	T_50_ (CH_3_OH) (°C)	DME Selectivity (%)	T_50_ (C_2_H_5_OH) (°C)	DEE Selectivity (%)	Reference
MCM-22_15	137	100	148	94	this work
MCM-36_15	138	100	151	94	this work
ITQ-2_15	144	100	149	96	this work
PCH-Al ^1^	239	100	238	75	[19]
PCH-Si ^2^	255	100	250	65	[19]
γ-Al_2_O_3_	289	100	308	72	[21]
Alophane ^3^	237	99	238	74	[21]
V-2 ^4^	344	92	301	28	[20]
PILC(C)-Al ^5^	225	100	220	57	[20]
Al-SBA-15 ^6^	225	100	-	-	[36]
Beta (Si/Al = 21)	160	100	-	-	[37]
Y (Si/Al = 16)	182	100	-	-	[37]
ZSM-5(Si/Al = 16.5)	205	100	-	-	[37]

^1^ Porous clay heterostructure obtained from montmorillonite intercalated with silica–alumina pillars; ^2^ Porous clay heterostructure obtained from montmorillonite intercalated with silica pillars; ^3^ Mineral allophane containing albite, illite, quartz, and crystobalite as impurities; ^4^ Vermiculite treated with a solution of HNO_3_(0.8 M) at 95 °C for 2 h; ^5^ Alumina pillared interlayered clay obtained from acid treated vermiculite (0.8 M HNO_3_, 95 °C, 2 h and washed with a solution of citric acid); ^6^ SBA-15 modified with aluminum oligocations.
